# Management of Pregnant Women after Bariatric Surgery

**DOI:** 10.1155/2018/4587064

**Published:** 2018-06-03

**Authors:** Jürgen Harreiter, Karin Schindler, Dagmar Bancher-Todesca, Christian Göbl, Felix Langer, Gerhard Prager, Alois Gessl, Michael Leutner, Bernhard Ludvik, Anton Luger, Alexandra Kautzky-Willer, Michael Krebs

**Affiliations:** ^1^Gender Medicine Unit, Division of Endocrinology and Metabolism, Department of Medicine III, Medical University Vienna, Vienna, Austria; ^2^Division of Endocrinology and Metabolism, Department of Medicine III, Medical University Vienna, Vienna, Austria; ^3^Division of Obstetrics and Feto-Maternal Medicine, Department of Obstetrics and Gynecology, Medical University of Vienna, Vienna, Austria; ^4^Department of Surgery, Medical University Vienna, Vienna, Austria; ^5^Medizinische Abteilung mit Endokrinologie, Diabetologie, Nephrologie, Krankenanstalt Rudolfstiftung, Vienna, Austria

## Abstract

The prevalence of obesity is growing worldwide, and strategies to overcome this epidemic need to be developed urgently. Bariatric surgery is a very effective treatment option to reduce excess weight and often performed in women of reproductive age. Weight loss influences fertility positively and can resolve hormonal imbalance. So far, guidelines suggest conceiving after losing maximum weight and thus recommend conception at least 12–24 months after surgery. As limited data of these suggestions exist, further evidence is urgently needed as well for weight gain in pregnancy. Oral glucose tolerance tests for the diagnosis of gestational diabetes mellitus (GDM) should not be performed after bariatric procedures due to potential hypoglycaemic adverse events and high variability of glucose levels after glucose load. This challenges the utility of the usual diagnostic criteria for GDM in accurate prediction of complications. Furthermore, recommendations on essential nutrient supplementation in pregnancy and lactation in women after bariatric surgery are scarce. In addition, nutritional deficiencies or daily intake recommendations in pregnant women after bariatric surgery are not well investigated. This review summarizes current evidence, proposes clinical recommendations in pregnant women after bariatric surgery, and highlights areas of lack of evidence and the resulting urgent need for more clinical investigations.

## 1. Introduction

Obesity is associated with higher rates of cardiometabolic comorbidities and mortality and is increasing worldwide since decades. Effective weight loss approaches are necessary to overcome the negative long-term effects of obesity. Among lifestyle and medical treatment, bariatric surgery is a commonly used method in severely obese patients, which was demonstrated to result in good weight loss outcome. Between 1998 and 2005, the numbers of bariatric surgeries have increased by 800% [1]. These surgeries are performed in about 80% in women and about half of them in women of reproductive age. A British Registry report indicates that 53% of surgeries are performed in women between 18 and 45 years of age, a significant underrepresentation of non-Caucasian women and one-third of women having menstrual dysfunction [2]. Pregnant women after bariatric surgery have to be controlled regularly by a specialized team with specialists of various fields familiar with the management after bariatric procedures. The special needs of these pregnant women are to be addressed individually [3]. Supplementation of vitamins, minerals, and trace elements after bariatric surgery as well as during pregnancy is essential to avoid deficiencies and further arising complications in mother and child. Data from the US suggest poor screening for any deficiency in less than half of the women, but higher rates in pregnancy [4]. According to a recent survey, high unawareness (<20%) regarding nutritional recommendations and control was found among obstetricians [3].

## 2. Methods

Peer-reviewed literature reporting about bariatric surgery and pregnancy was critically examined. Using Medical Subject Heading (MESH) search terms in the PubMed database restricted to humans only and no time limit gave a result of 298 relevant articles from 1986 up to April 2018. Terms used in the search were obesity, bariatric surgery, and pregnancy linked with Boolean search operators. All abstracts were searched for relevant information about topics around the management of pregnant women after bariatric surgery. Pertinent literature was carefully examined, and further hand search was carried out to identify additional literature of relevance from the reference lists. Forward and backward literature searches were performed for highly important articles only and for literature regarding nutritional recommendations, deficiencies, and supplementation in pregnant women after bariatric surgery. Additionally, the homepages and published materials/guidelines of relevant national and international health, obesity, diabetes, surgical, and nutrition associations were searched to find further relevant information. The high variability of all sources of information led to the decision to perform a descriptive synthesis approach of the final 110 articles cited in our review. Level of evidence and grade of recommendation were assigned with the help of existing comprehensive reviews [5, 6].

## 3. Results

### 3.1. Obesity and Fertility

Obese women planning to conceive have a lower likelihood to become pregnant compared to lean women [7]. This decrease in fertility is primarily based on menstrual irregularities or anovulation. Overweight and obesity are associated with menstrual irregularities in a cross-sectional study reporting 30–47% of overweight/obese women presenting with menstrual anomalies, which correlates with increasing BMI [8]. Dissatisfaction with their sexual life was reported in about 50% of severely obese female and male patients [9]. Next to these findings, a longer duration of menstrual cycles was observed [10]. This might be caused by increased circulatory androgen concentrations (testosterone, DHEA-S), which are raised due to decreased hepatic SHBG production. Hepatic SHBG production is negatively influenced by hyperinsulinemia, which is more prevalent in obesity [10, 11]. Additionally, hyperinsulinemia triggers LH-mediated androgen production in ovarian theca cells [12]. These factors and their pleiotropic effects on other hormones cause an imbalance resulting in infertility. After weight loss surgery, a steep increase of SHBG and decline of testosterone, androstenedione, and DHEA-S levels were observed in obese women, which might help to overcome menstrual anomalies and infertility [13]. The quality of sexual life improves significantly over time in men and women after weight loss surgery due to significant increases in body image satisfaction [14].

### 3.2. Fertility after Bariatric Surgery

Clear decreases after bariatric surgery in prevalence of T2DM, PCOS, and menstrual irregularities were observed. Effects of bariatric surgery on fertility are mostly reported in small studies including small number of participants. Thus, evidence is limited and further studies are necessary to assess the effects of bariatric surgery on fertility and hormonal parameters. Reviews [1, 15] show a positive effect of weight loss through bariatric surgery on hormonal parameters with significant decreases in estrogen and testosterone and increases in FSH, LH, and SHBG. Furthermore, a decrease in TSH levels was observed, with no changes in free T4, increases in free cortisol, and decreases in cortisol binding protein [15]. Females after bariatric surgery reported normalization of menstrual cycles, regular ovulation, and more often spontaneous conception [1, 15]. A recent systematic review investigating gonadal dysfunction in obese patients and resolution of gonadal function after bariatric surgery found that 36% (95% CI 22–50) of women had PCOS. This resolved in 96% (95% CI 89–100) of women after surgical intervention with reduction of signs of hyperandrogenemia and amelioration of menstrual anomalies due to weight loss surgery [16].

### 3.3. Planned Pregnancy

Obese women in reproductive age aiming to perform bariatric surgery need to be informed that after bariatric surgery, the probability to get pregnant without sufficient contraception is increased. Rapid weight loss after bariatric surgery may reduce symptoms such as anovulation or cycle irregularities. Thus, in reproductive age, pregnancies are not recommended shortly after bariatric surgery and need to be planned after the phase of maximum weight loss, as short- and long-term consequences of rapid weight loss and potential micronutritional deficiencies on the offspring are not well investigated. At least 12 to 18 months and in some publications up to 24 months or until stabilization of weight after surgery are recommended between surgery and conception (evidence level 3, grade of recommendation D) [5–7, 17–19]. Individual progress of weight loss and weight stabilization needs to be addressed. When planning a pregnancy, regular control intervals with consultation of different specialities are recommended after bariatric surgery (Table 1). Limited evidence is available for conception before 12-month time lapse after surgery, but studies have shown comparable pregnancy outcomes comparing pregnant women before 12 months and thereafter [20]. Further studies demonstrated comparable rates of gestational diabetes mellitus, pregnancy-induced hypertension, birth weight, intrauterine growth restriction (IUGR), or small-for-gestational age (SGA) offspring [21]. However, little evidence exists about these aspects in women after bariatric surgery. After fasting in pregnancy ketonemia, increased urinary nitrogen excretion and decreased gluconeogenic amino acid production were reported, and due to physiological increases of insulin resistance in pregnancy, higher risk of ketonemia and ketonuria was suspected [22]. Thus, weight loss in pregnancy, especially shortly after bariatric procedures, might cause significant maternal metabolic changes, which potentially affect fetal development (growth, biometry, and malformation) or future disposition for healthy development and disease in offspring (neurocognitive, cardiovascular, and metabolic), which are underinvestigated so far.

If pregnancy occurs within time of maximum weight loss, short control intervals of mother and offspring are recommended with regular endocrine and metabolic examinations and documentation of general health appearance, weight curve, blood parameters, nutritional behaviour and nutritional intake, and advice (if necessary, sufficient supplementation with minerals, trace elements, and vitamins has to be prescribed; see below sections). Regular obstetric investigations monitoring fetal biometry with documentation of growth and well-being of the fetus, general health appearance of the mother, and planning of further pregnancy and birth modalities are necessary. If surgery-related complications cannot be ruled out, metabolic surgeons need to be involved early. It is recommended to plan delivery in a tertiary care centre with experienced interdisciplinary teams and the availability of a neonatal intensive care unit.

Women in reproductive age after bariatric surgery should be informed about the importance of nutritional supplementation in case of an emerging pregnancy and the need of compliance regarding intake and examinations. Pregnancy planning and waiting until time after maximum weight loss and optimization of nutritional supply (e.g., folic acid) before conception is favourable and should be recommended to all women undergoing bariatric surgery in reproductive age.

### 3.4. Contraception

A recent study reported that more than 4% of women tried to conceive in the first postsurgical year and another 41% had unprotected sexual intercourse during this time [23]. This study uncovers the need of more information about postsurgical contraception and time lapse between surgery and conception towards women after bariatric surgery. Oral contraception may not provide sufficient protection after bariatric surgery (especially in gastric bypass procedures). Malabsorption and complications such as vomiting and diarrhoea may cause limited effectiveness [19, 24]. There is lack of evidence due to low number of studies performed so far. A review summarizing five studies giving limited evidence concludes no reduction of effectiveness of oral contraceptives after bariatric surgery [25]. However, current ACOG guidelines recommend cautiousness, as in a few cases, pregnancies occurred unplanned. Lower absorption rates are suspected [19]. In particular, in RYGB and malabsorptive procedures, other contraceptive methods than oral ones are recommended (evidence level 3, grade of recommendation D) [5, 6, 19]. Undoubtedly, further evidence is urgently needed to increase knowledge about effectiveness of oral contraceptives after bariatric surgery.

### 3.5. Pregnancy

In case of pregnancy after bariatric procedures, follow-up visits and examinations have to be performed in short intervals (Table 1). If controls are missed or not scheduled, higher risk of persistent vomiting, gastrointestinal bleeding, anaemia, placental vascular disease, fetal neural tube defects, intrauterine growth retardation, or even miscarriage is reported [18]. In women with LABG, adaption might be necessary already starting from first trimester to prevent complications such as vomiting (evidence level 2, grade of recommendation B) [21, 26]. This so-called active gastric band management must be performed by an experienced surgeon.

Dietary advice and monitoring of food intake at regular intervals performed by trained dieticians with special knowledge of needs after bariatric procedures and experience in advising pregnant women are needed (evidence level 1, grade of recommendation A) [6, 19]. If possible, appointments should be performed before a pregnancy and at least every trimester in pregnancy and if necessary even at closer intervals (Table 1).

### 3.6. Examinations in Pregnancy

Pregnant women after bariatric surgery need to undergo regular examinations at least every trimester at specialized facilities (evidence level 3, grade of recommendation C) [5, 6, 19]. It is important to check nutritional state and recognize nutritional deficiencies at an early stage and try to prevent them [6]. Examinations also include blood sampling which should be performed at least once per trimester and include full blood count, clinical chemistry, coagulation, vitamins A, D, E, K, B12, iron status, folic acid, parathyroid hormone and protein, albumin, A1c, glucose, and TSH [6, 27]. According to Mechanik et al., several parameters have to be checked or ruled out which are included in Table 1 [6]. Table 1 provides further important aspects, which need to be considered.

### 3.7. Pregnancy and Obstetric Management

In general, bariatric procedures should not be regarded as a contraindication to deliver naturally [19, 28]. Nevertheless, increased rates of C-section in operated women are reported with some recent publications showing no differences [28]. However, huge variations in C-section rates were found in the literature ranging from about 18 to 60% section rate in operated women compared with 14–29% in control groups [28]. Explanations were found in a recent review, which discusses former C-section as the main issue, next to other aspects as maternal obesity, selection of the mother, the fetal position, and perceptions of treating clinicians [28].

### 3.8. Diagnosis of Gestational Diabetes Mellitus

Several studies have demonstrated that the prevalence of GDM decreases after bariatric procedures [29–31]. On the contrary, obese women have high risk of GDM throughout pregnancy: up to nearly 40%, with high incidences documented already in early pregnancy, and features of the MetSy, which might contribute to pregnancy complications [32, 33]. So far, the procedures that should be employed to diagnose gestational diabetes are unclear in pregnancies after bariatric surgery as several problems may arise. Depending on the type of bariatric surgery (e.g., RYGB), fast glucose absorption during an OGTT might lead to severe postabsortive hypoglycaemia [7]. Recent evidence demonstrates difficulties in the interpretation of OGTT results as plasma glucose concentrations after oral glucose load are altered following gastric bypass and characterized by rapidly changing glucose levels as well as high risk for reactive hypoglycaemic events following glucose load [34–37]. This might lead to misinterpretation of postprandial glucose levels as one-hour levels misleadingly appear too high, and two-hour levels appear too low, and thus, diagnostic alternatives to define impaired glucose tolerance in pregnancies affected by metabolic surgery need to be found. Moreover, testing of GDM might be related to serious adverse events as the dumping syndrome might occur especially in women after RYGB, omega loop, or sleeve gastrectomy [36]. Thus, no recommendations exist so far, which advise to perform an OGTT between 24 and 28 gestational weeks to diagnose gestational diabetes mellitus in women after bariatric surgery. As an alternative to an OGTT, ACOG [19] advised to perform home glucose monitoring for several days (i.e., about one week) with measurement of fasting and postprandial glucose levels and additional measurements if symptomatic (hyper- or hypoglycaemic event), which was also recommended by Adam et al. [36] between 24 and 28 weeks of gestation (evidence level 3, grade of recommendation D). Another alternative is to measure capillary glucose from 14 to 16 weeks of gestation with continuation throughout pregnancy [36]. Continuous glucose monitoring (CGM) or flash glucose monitoring (FGM) systems are an upcoming tool and of special interest as more and more easily implementable devices are available nowadays. These devices might be especially helpful in women with hypo- or hyperglycaemia at regular intervals and can help to evaluate glycaemic control. FGM was found to be safe and accurate in pregnant women with diabetes [38]. A recently published case report described the successful use of FGM in a pregnancy after RYGB complicated by GDM and nocturnal hypoglycaemia [39]. However, further studies are necessary to evaluate safety and accuracy of FGM in pregnant women after bariatric surgery. So far, alternative diagnostic methods have been described, but validated diagnostic criteria are not available. Diagnostic criteria to determine GDM in pregnancies and overt diabetes in early pregnancy following bariatric surgery are shown in Table 2 (evidence level 4, grade of recommendation D). Overt diabetes in early pregnancy is diagnosed as recommended for nonpregnant individuals after bariatric surgery [40]. In pregnancy, postprandial glucose levels are important for GDM diagnosis and treatment initiation as they are associated with fetal hyperinsulinemia, fetal growth, birth weight, and abdominal circumference. Due to changes in glucose absorption after bariatric procedures, rapid postprandial plasma glucose increases followed by rapid decreases and risk for dumping syndrome occur in many patients. In pregnant women after RYGB, postprandial hypoglycaemia was reported in nearly 55% up to 90% of women after a 75 g OGTT between 24 and 28 weeks of gestation [34, 37]. Furthermore, higher incidence of SGA offspring and associations of postprandial glucose nadir with fetal growth were reported [34, 37]. Thus, the use of elevated 1 h glucose values solely for diagnosis and initiation of insulin treatment is not advisable, and fasting and 2 h postprandial glucose values seem to be better and safer parameters to base upon diagnostic and treatment decisions (evidence level 4, grade of recommendation D). A similar constellation exists in the diagnosis of overt diabetes before 20 weeks of gestation, which should be based upon fasting values and HbA1c. If a dumping syndrome is suspected, additional postprandial measurements beyond the 2 h measurement are necessary and recommended. In case of a diagnosis of gestational diabetes mellitus or overt diabetes in pregnancy, the controls need to be intensified and individual therapeutic approaches need to be developed. Similar perinatal outcomes and glycaemic control are reported in women with GDM after bariatric surgery and women with GDM without surgery [7].

The management of early or late dumping syndrome, as well as other complications (e.g., diarrhoea, flatulence, constipation, dysphagia, vomiting, food intolerance, and dehydration), is explained in detail elsewhere [42, 43]. Shortly, in early dumping, the amount of food per portion needs to be reduced and split into at least six meals a day [42]. A delay of liquid intake of at least 30 minutes after the meal is recommended. Management of late dumping includes the avoidance of rapidly absorbable and refined carbohydrates [42]. A case report demonstrated the beneficial use of acarbose in pregnant women after RYGB with severe progressive hypoglycaemic events as other interventions were not successful [42]. A significant reduction of postprandial hypoglycaemic events and the birth of a healthy girl at term with normal development were reported.

### 3.9. Weight Gain in Pregnancy

Weight gain in pregnancy should follow IOM recommendations, which are shown in Table 3 as no other evidence regarding weight gain recommendations in bariatric pregnancies exist (evidence level 4, grade of recommendation D) [19, 44]. Data so far reported lower weight gain in pregnancy after bariatric surgery compared with nonoperated obese women matched for BMI [21]. In a recent small study including women after RYGB, a mean gestational weight gain of 3.8 ± 12 kg was found and no differences in gestational weight gain were found when comparing women who became pregnant before or after the first year after surgery [45]. If weight gain in pregnancy does not follow IOM recommendations, more intense control intervals based on individual need have to be considered. Time lapse between surgery and conception might affect gestational weight gain and postpartum weight loss [7]. Fetal growth needs to be monitored in narrow intervals. A recent systematic review provides information that weight gain below or above the IOM recommendations for the respective weight class was associated with adverse perinatal outcomes [46]. In women with weight gain below the IOM recommendations, higher risk for SGA and preterm birth was reported.

### 3.10. Outcome

In general, a higher risk in obese pregnant women is well known for gestational diabetes, hypertension, preeclampsia, miscarriage, caesarean section, and stillbirth. In postbariatric surgery pregnancies, decreased risk for maternal complications was reported with approximation to risks of normal-weight women and improved neonatal outcomes compared with obese women without intervention [1, 5, 7, 18, 47]. In pregnancy, lower risk of gestational diabetes mellitus, hypertension, preeclampsia, and miscarriage was detected in operated women compared with obese women [17, 21, 28, 47]. Lower rates of preterm birth were reported, but also conflicting data exist with some studies reporting higher rates of LGA or SGA infants [7, 21, 25, 28]. Most studies report no differences in prematurity rate and perinatal death [21]. Recent results from a Swedish study representing more than 625,000 singleton pregnancies corroborate these results and demonstrate lower risk for GDM (OR 1.9% versus 6.8%; 95% CI 0.13; 0.47; *p* < 0.001) and lower pregnancy duration (273.0 versus 277.5 days; 95% CI; −2.9; −6.0; *p* < 0.001) [29]. However, diagnostic criteria for GDM in this study remain problematic. GDM was diagnosed based on the results of a 75 g oral glucose tolerance test according to the standard national criteria: if fasting plasma glucose exceeded 7.0 mmol/l (126 mg/dl) or 2-hour plasma glucose exceeded 10.0 mmol/l (180 mg/dl) [29]. In case of high risk of hypoglycaemia, fasting glucose and preprandial and postprandial glucose values were used for diagnosis instead [29]. Lower risk for LGA births (OR 8.6% versus 22.4%; 95% CI 0.24–0.44; *p* < 0.001) and higher risk for SGA (OR 15.6% versus 7.6%; 95% CI 1.64–2.95; *p* < 0.001) but also potentially higher intrauterine and neonatal mortality risk (OR 2.39; 95% CI 0.98–5.85; *p*=0.06) were reported [29]. An Israeli observational study found significant reduction in diabetes mellitus (OR 0.6; 95% CI 0.4–0.9; *p*=0.009), hypertensive disorders (OR 0.4; 95% CI 0.3–0.6; *p* < 0.001), preeclampsia (OR 0.2; 95% CI 0.1–0.7; *p*=0.005), anaemia (OR 0.7; 95% CI 0.5–0.9; *p*=0.014), and fetal macrosomia (OR 0.5; 95% CI 0.2–0.9; *p*=0.033) in women after bariatric surgery [48]. This study found no significant differences in GDM risk, but diagnostic criteria were not reported. A French observational study found significantly lower weight gain in women after LABG compared to obese women (5.5 versus 7.1 kg; *p* < 0.05) and lower risk for GDM (0 versus 22.1%; *p* < 0.05), preeclampsia (0 versus 3.1%; *p* < 0.05), low birth weight (7.7 versus 10.6%; *p* < 0.05), fetal macrosomia (7.7 versus 14.6; *p* < 0.05), and caesarean section (15.3 versus 34.4, *p* < 0.01). No differences were found in other neonatal outcomes. Diagnostic criteria for GDM were not published either in this study [49]. An American study investigating women before and after bariatric surgery found lower GDM incidence (OR 0.23; 95% CI 0.15–0.36) and lower risk for caesarean section (OR 0.53; 95% CI 0.39–0.72) in women after bariatric procedures [30]. The ICD-9 code for GDM was used to define GDM in this study. A recently published meta-analysis reporting maternal and neonatal outcomes of 20 cohort studies and including about 2.8 million subjects matched for presurgery body mass index found lower maternal risk for GDM (OR 0.20; 95% CI 0.11–0.37; number needed to benefit (NNTB) 5), hypertension (OR 0.38; 95% CI 0.19–0.76; NNTB 11), hypertensive disorders (OR 0.38; 95% CI 0.27–0.53; NNTB 8), postpartum hemorrhage (OR 0.32; 95% CI 0.08–1.37; NNTB 21), and caesarean section (OR 0.50; 95% CI 0.38–0.67; NNTB 9) [50]. Diagnostic criteria of GDM were not specified in this meta-analysis. Furthermore, lower risk for LGA (OR 0.31; 95% CI 0.17–0.59; NNTB 6) and higher risk for SGA (OR 2.16; 95% CI 1.34–3.48; number needed to harm (NNTH) 21), IUGR (OR 2.16; 95% CI 1.34–3.48; NNTH 66), and preterm deliveries (OR 1.35 95% CI 1.02–1.79; NNTH 35) were reported. No differences in congenital malformations between obese women and women after bariatric surgery were reported [50]. However, further research is necessary to evaluate the risk of congenital malformation as case reports have reported increased risk of neural tube defects after gastric bypass surgery [51, 52].

Long-term outcomes revealed that offspring of women after biliopancreatic diversion had lower overweight and obesity risk reduced to population risk up to 18 years after birth and no increase in underweight, better insulin sensitivity, lipid metabolism and ghrelin levels, lower inflammatory parameters and leptin levels, and less hypertension compared to offspring of nonoperated women [53, 54]. However, age differences between groups (10 versus 16 years) need to be considered as a relevant confounder [53]. Interestingly, in a cohort of siblings born before and after biliopancreatic diversion of their mother, weight was comparable at ages 1 and 6, but significantly higher rates of overweight/obesity at the age of 12 years were detected in siblings born before bariatric surgery of the mother [55], while other studies do not report any differences after birth up to the age of ten years or preschool age [56, 57]. However, during pregnancy, positive associations were observed between differences in gestational weight gain and sibling's birth weight [57]. Significant differences in DNA methylation were found in 5698 genes between offspring of women before and after bariatric procedures [58]. Metabolic improvement found in offspring after surgical procedures was correlating with methylation patterns in genes involved in cardiometabolic pathways, which clearly demonstrates the effect of maternal treatment of obesity on cardiometabolic parameters of the offspring at both epigenetic and transcriptional levels [58]. However, a case-control study investigating micronutrient deficiencies in 56 neonates of mothers with RYGB found higher rates of decreased cord blood levels below the 2.5 percentile for calcium, zinc, iron, and vitamin A and above the 97.5 percentile for magnesium, vitamin E, D, and B12 in RYGB offspring [59]. In a follow-up of offspring with a mean age of 46 months of women with gastric bypass, inadequate fibre intake in all children and deficiencies in calcium, vitamin A, and folic acid were found [60].

### 3.11. Abdominal Pain and Surgical Complications

During pregnancy after bariatric surgery, complications including intestinal obstructions or hernia, gastric ulcer, band, or staple line complications have been reported [21], which all need fast reaction to minimize maternal and fetal risks. Strong persistent abdominal pain, excess vomiting, and persistent nausea necessitate urgent consultation of an experienced metabolic surgeon (evidence level 3, grade of recommendation D). A Swedish cohort study identified significant higher risk of abdominal surgery in pregnancy [61]. Higher rates of laparotomies (1.5% versus 0.1%; OR 11.3; 95% CI 6.9–18.5) and higher rates of intestinal obstruction (1.5% versus 0.02%; OR 34.3; 95% CI 11.9–98.7) were reported. In case series, small intestinal obstructions or inner hernias in pregnancy were described [25]. In a case of persisting vomiting, intravenous supplementation of vitamins and/or trace elements together with fluid replacement needs to be considered. In particular, vitamin B1 (thiamine) deficiency needs to be considered as patients after RYGB and BPD-DS are at higher risk, and in pregnancy, hyperemesis gravidarum might aggravate this condition [62]. Symptoms of thiamine deficiency are Wernicke encephalopathy, oculomotor dysfunction, and gait ataxia. If Wernicke encephalopathy is suspected, the administration of intravenous solutions containing glucose may further deplete the remaining available thiamine and precipitate Korsakoff's syndrome [62]. In case of thiamine deficiency, intravenous thiamine infusion with 100 mg thiamine followed by consecutive intramuscular injection (100 mg/day for 5 days) and oral maintenance (50–100 mg/day) should be applied [62]. The application of oral antibiotics (recommended in pregnancy: amoxicillin for 7–10 days per month over two months) is recommended according to Lakhani et al. [63], who hypothesized small intestinal bacterial overgrowth due to alterations in gut microbiome following bariatric surgery as a cause for thiamine deficiency.

After insufficient long-term nutrient intake, the reinstitution of nutrient intake should be performed gradually and preferably in an inpatient setting under close monitoring of electrolytes including potassium and phosphorous, since a potentially life-threatening refeeding syndrome might occur. If a gastric band was implanted, a metabolic surgeon needs to assess a relaxation of the gastric band already in early pregnancy. In earlier studies, band migration with consequent complications (vomiting, disturbances in electrolyte and fluid balance, and band leakage) was described in nearly 29% [64]. A systematic review reported five neonatal and three maternal deaths and the necessity of acute surgical intervention in 20 cases in pregnancy with the majority of intervention due to internal hernia after RYGB [1]. Further, 23 women requiring urgent surgical intervention due to internal hernia but no death were reported in another study [65]. A recent case report describes acute bowel ischemia following thrombosis of the superior mesenteric artery in pregnant women after RYGB with loss of the fetus, several acute laparotomies, and subtotal enterectomy, preserving the first 20 cm of the jejunum [66].

### 3.12. Supplements during Pregnancy

Regular follow-ups to detect nutritional deficiencies before pregnancy and during pregnancy at least every trimester are recommended (Table 1). After bariatric surgery, micronutrient supplementation should be provided to all pregnant women (evidence level 3, grade of recommendation D). A recent systematic review summarizes several relevant cohort studies and case reports describing micronutritional deficiencies in pregnancies after bariatric surgery and found associations of vitamin K, A, B12, folate acid, and iron depletion with maternal and fetal complications (see sections below), but not for other micronutrients as calcium, zinc, magnesium, iodine, or copper [27]. The authors clearly concluded the need of further studies in this field as the information collected about subsequent adverse events concerning mother or child is weak and inconclusive [27]. The multicentre prospective cohort study AURORA will contribute to elucidate our knowledge about various aspects of women who underwent bariatric surgery including micronutrient deficiencies before, during, and after pregnancy [67].

### 3.12.1. Protein

According to German-Austrian-Swiss (D-A-CH) nutritional recommendations for normal pregnancy, daily protein intake is recommended with 0.9g protein per kilogram body weight in the second trimester and 1.0 g/kg in the third trimester [68]. Calculations are always based on normal weight (also in overweight/obese patients). During lactation, 1.2 g/kg is recommended [68]. Recommendations of daily intake for protein in pregnancy after bariatric surgery are not available and might depend on the type of bariatric surgery and time lapse from surgery.

### 3.12.2. Micronutrients

*(1) Iron*. Due to expansion of the blood volume, iron demand increases from 15 mg/d to 30 mg/d. Haemoglobin levels decrease physiologically. According to the WHO criteria, anaemia is defined as haemoglobin level below 110 g/l. The iron status should be examined at regular intervals, as well as haemoglobin levels, which determine the intensity of iron supplementation. Treatment of iron deficiency should start orally. Intravenous iron is not recommended in the first trimester [69]. Oral calcium and iron supplements interact and should not be taken in combination. Interestingly, in pregnancies with RYGB longer than four years time to conception, significantly lower haemoglobin levels (9.6 versus 11.1 g/dl; *p*=0.047) and higher need of intravenous iron substitution or packed red cell transfusion were identified (30.8% versus 0%; *p*=0.026) compared to women with less than 4 years time to conception [70]. Besides anaemia, no other significant complications in mother or child were reported [27].

*(2) Calcium*. Calcium homeostasis is strongly influenced by bariatric surgery as well as pregnancy. An acidic environment is required to allow absorption of calcium. Throughout pregnancy and lactation, a higher calcium demand is known, which may be critical for women after a bariatric procedure, regarding bone density and dental state [71]. Especially in the last trimester, a significant transfer from the mother to the fetus is observed to increase fetal skeletal mineralization. Thus, calcium is mobilized from the maternal calcium reservoir, which is mainly bone, and renal calcium retention, which increases risk for osteoporosis [71]. Higher calcium doses in pregnancies after bariatric surgery are recommended compared with normal pregnancies. Calcium deficiency was reported in 15.2% in the first and second trimesters and 20% in the third trimester in pregnant women after RYGB [72]. PTH excursions were found in 19.6, 30.4, and 32.6% from first to third trimester, respectively.

*(3) Magnesium*. Nocturnal calf cramps occur in 5–30% of pregnant women. They are associated with low magnesium levels. These can be well treated by oral magnesium supplements. In addition, it is useful in the prevention of muscular contractions of the uterus [73, 74]. High doses of magnesium can cause osmotic diarrhoea.

*(4) Zinc*. Low zinc levels, which also occur in pregnant women without bariatric surgery, are associated with early childbirth, low birth weight, and spina bifida. During lactation, eczema, dermatitis, and failure to thrive were reported in the offspring [75]. In order to prevent subsequent copper deficiency, at least 1 mg of copper should be given per each 8–15 mg of zinc substitution [43]. Zinc inadequacy in pregnant women after RYGB was reported in 20%, with no associations to birth weight or maternal anthropometry [76].

*(5) Iodine*. Iodine deficiency is common in Middle Europe, and a recent analysis demonstrates iodine deficiency even in normal pregnancies [77]. Only 13.8% of the participating women were in the recommended range of 150–249 *µ*g/l iodine urinary concentration despite commercially available iodized table salt. The upper urinary concentration of 250 *µ*g should not be exceeded because of the significant association with subclinical hypothyroidism, whereas the WHO recommends not to exceed a urinary iodine concentration of 500 *µ*g in pregnant women [78]. After bariatric surgery, limited resorption in women planning to become pregnant or in pregnant women might be associated with lower urinary iodine concentrations. Especially after malabsorptive interventions, evidence is scarce, particularly considering resorption of iodine happens in the stomach and small intestine. In nonpregnant subjects after malabsorptive interventions, increased urinary iodine concentration was found 3 to 18 months after bariatric surgery [79, 80] Furthermore, no iodine deficiency was identified ten years after gastric bypass or vertical banded gastroplasty [81]. So far, no studies reported maternal or fetal adverse events in pregnancy due to iron, calcium, magnesium, zinc, or iodine deficiency after bariatric procedures [27].

#### 3.12.3. Vitamins

In general, substitution using vitamin supplements is recommended in pregnancy as well as after bariatric surgery, especially in case of deficiencies identified (evidence level 3, grade of recommendation D). Multivitamin preparations also for use in pregnancy may contain vitamin A or retinol equivalents and have to be prescribed cautiously because of potential teratogenicity in high doses.  Table 4 shows nutritional intake recommendations in pregnancy.

*(1) Vitamin D*. In healthy adults, a daily vitamin D intake of 800 IU is recommended following D-A-CH recommendations [68]. The target level is a 25(OH)D serum concentration of above 50 nmol/l (20 ng/mL). The Endocrine Society recommends a maximal dose of 4000 IU/d in pregnancy or when planning to get pregnant [83]. In postbariatric populations, doses up to 6000 IU/d are discussed for nonpregnant women [6]. A study evaluating vitamin D status and its relations with ionic calcium and parathyroid hormone (PTH) in pregnant women after RYGB found vitamin D deficiency (≤20 ng/mL) or insufficiency (>20–30 ng/mL) above 70% in all trimesters [72]. Negative correlations between calcium and PTH as well as an association of vitamin D with higher risk of urinary tract infection were reported.

*(2) Folic Acid*. Women planning to become pregnant should substitute folic acid after stabilization of their body weight. The substitution should start at least four weeks before conception and continue in pregnancy. There is no evidence on higher demands of folic acid in women after bariatric surgery [19]. A daily intake of 0.4 mg folic acid is recommended. Prevalence of folic acid deficiency was reported in 0–16% of pregnant women with bariatric procedures [84, 85]. Deficiencies of folic acid in and before pregnancy are associated with higher risk of neural tube defects. In a case series of three patients with no preconceptional nutritional counselling and poor postsurgical surveillance, severe neural tube anomalies were reported [86]. Thus, higher doses of folic acid up to 5 mg might be needed due to higher demands and deficiencies reported after bariatric surgery, which are also recommended in women with type 2 diabetes mellitus and body mass index above 30 kg/m^2^ until twelve weeks of gestation [5].

*(3) Vitamin B12*. Vitamin B12 levels should be regularly controlled. In case of deficiency, vitamin B12 should be administered parenterally or orally if locally available. Prevalence of vitamin B12 deficiency was reported in about 50% of pregnant women with bariatric procedures [84, 85]. Neonatal vitamin B12 deficiency may cause irreversible neurologic defects and thus needs to be detected early [27, 87].

*(4) Vitamin A*. In the literature, recommended vitamin A doses are divergent. During pregnancy, the D-A-CH society recommends a retinol equivalent of 1100 *µ*g (i.e., 3666 IU) per day from the fourth month of gestation onwards until the end of pregnancy. An upper limit of 5000 IU (1600 *µ*g retinol equivalent) with inclusion of different vitamin A isoforms (retinol, retinol ester, *β*-carotene) in nutrition is described in American literature to prevent malformations [19, 75, 88]. The EFSA (European Food Safety Agency) stated in their most recent recommendation a tolerable upper intake level of 3000 *µ*g/d retinol equivalent in pregnancy [89]. In women planning a pregnancy or pregnant women, the *β*-carotene form of vitamin A is recommended over retinol [43]. More than half of pregnant women with bariatric surgery were found to be deficient in vitamin A levels [84]. This was corroborated by two Brazilian studies evaluating vitamin A status among pregnant women after RYGB, which found inadequate serum retinol or *β*-carotene concentrations in about 60% of women during and after pregnancy with higher rates of symptoms (night blindness) reported (57% during pregnancy) [76, 90]. Significant associations of vitamin A deficiency with urinary tract infection and dumping syndrome were found. A case of severe maternal vitamin A deficiency after biliopancreatic diversion with premature birth and ophthalmologic and renal malformations was reported [91].

*(5) Vitamin E*. The elimination of free radicals is associated with vitamin E. The D-A-CH society recommends a daily intake of 13 mg tocopherol equivalent (=19.4 IU) [21], and the EFSA recommends a daily intake of 11 mg for women with no additional need in pregnant or lactating women and a 300 mg/d (=447 IU) upper tolerable intake level [92].

*(6) Vitamin K*. The D-A-CH society recommends a daily vitamin K intake of 60 *µ*g. Due to lack of evidence, the EFSA could not define a tolerable upper intake level for vitamin K [93]. A daily intake of 70 *µ*g phylloquinone is recommended [93]. After bariatric surgery, vitamin K shows reduced absorption and consequently transfers across the placenta. Thus, monitoring might be useful. Either direct measurement of vitamin K or indirect measurement of prothrombin time is possible. Deficiencies are reported in a high proportion of pregnant women after bariatric procedures reaching nearly 90% in first trimester and about half of the women at birth. In one study, no complications were reported [94]. However, five cases of intracranial bleeding associated with vitamin K deficiency and malformations have been described [95]. Furthermore, after biliopancreatic diversion, a case of vitamin K deficiency with maternal coagulopathy and vaginal hemorrhage and fetal hypocoagulability was reported [96]. These are rare but severe complications. Chronic complications including psychomotor and mental retardation from bleedings or even neonatal death were reported [27].

#### 3.13. During Lactation

The lactational phase is a very important period for the development of the offspring. During lactation, regular examinations in 3-month intervals are recommended in women after bariatric surgery. In case of hyperglycaemia in pregnancy, fasting glucose or HbA1c control is advised four to twelve weeks after birth to document impaired glycaemic control postpartum. An oral glucose tolerance test should not be performed due to high risk of hypoglycaemic adverse events and high variability of glucose levels postprandially. Fasting glucose and HbA1c are recommended to be controlled and indicate a diagnosis of diabetes if they exceed 126 mg/dl or 6.5% (5.6 mmol) [40] (evidence level 4, grade of recommendation D). However, high variability of glucose levels was documented in postprandial glucose studied by continuous glucose monitoring (CGMS) [97]. Furthermore, CGMS detected high risk of postprandial hyperglycaemia in patients who were thought to have diabetes remission after surgery following actual guideline recommendations and had shown a normal fasting glucose and HbA1c [40, 97]. Thus, capillary home blood glucose monitoring with several time points postprandially, CGMS, or FGM may be offered additionally to collect fasting and postprandial glucose levels over a few days in case of uncertainty. Micronutrient deficiencies have to be identified with control of parameters as described above. Regular examinations of the newborn and examinations of the offspring in general are highly recommended.

The WHO recommends for all mothers to exclusively breastfeed until 6 months after birth. There are no exceptions known for mothers after bariatric surgery; however, concerns regarding micronutrient and vitamin deficiencies in exclusively breastfed offspring have been raised (evidence level 4, grade of recommendation D) [43]. Breastfeeding mothers need to be controlled and sufficiently supplemented because the maternal intake of nutrients has strong influence on the quality of the breast milk delivered to the offspring [98]. A recent study found significantly higher fat, energy, and a slightly higher carbohydrate breast milk content as well as no correlations between milk macronutrient composition and maternal diet in mothers after bariatric surgery compared with nonoperated controls [99]. Malnutrition of the mother can potentially cause undernourishment of the breastfed offspring. A sufficient vitamin and mineral supply is recommended. Especially vitamin B deficiency can cause megaloblastic anaemia and developmental delay in the offspring [43, 98, 100, 101]. Vitamin B12 deficiency in exclusively breastfed infants presenting with pancytopenia and long-term developmental delay was reported previously after RYGB caused by maternal vitamin B12 deficiency in breast milk [100, 101]. Calcium deficiency may lead to reduced calcium secretion in the breast milk and might cause undersupply and insufficient mineralization of the fetal bones [98]. However, evidence does not exist regarding supplementation of vitamins and trace elements after bariatric surgery during lactational period. The suggestions are derived from the D-A-CH guidelines for daily advised intake of nutrients in lactational period in women with no bariatric procedure as shown in Table 5 [68]. Furthermore, the upper intake levels and recommendations after bariatric surgery are shown.

## 4. Conclusions

The knowledge about the management of women after bariatric surgery in and around pregnancy is growing but consists mostly of data derived from retrospective studies or derived from few cohort studies and several case reports describing complications. However, there is little evidence in various important fields and aspects around pregnant women who underwent bariatric surgery with regard to ideal time of pregnancy after surgery, diagnostic criteria and best ways and methods to identify GDM, diabetes in pregnancy, and treatment goals after diagnosis. For pregnant women after metabolic surgery, further information on optimal weight gain in pregnancy, potential lack of several nutrients and nutritional intake recommendations in pregnancy and lactation, effects of nutritional deficiencies on fetal development, and long-term consequences in offspring is urgently needed and is of high scientific and clinical interest facing growing surgery numbers in women of reproductive age. These many uncertainties demonstrate a clear need of prospective studies focusing on filling these remaining gaps in knowledge. Based on the results and data collected in this review, further approaches and studies need to be conducted.

Pregnancies after bariatric surgery need to be considered as high-risk pregnancies with many potential complications, which may arise during pregnancy. These complications need to be accounted promptly to prevent acute or chronic complications in women with bariatric surgery or their offspring. Thus, care of these patients needs to be organized in an individual setting in a multilateral cooperation of various medical disciplines in specialized centres.

## Figures and Tables

**Table 1:** Parameters and aspects recommended to control in women planning a pregnancy and during pregnancies after bariatric surgery.

History of preexisting comorbidities such as diabetes mellitus, retinopathy, nephropathy, neuropathy, or hypertension
Regular follow-up visits after bariatric surgery are recommended when planning a pregnancy:
(i) Nutritional counselling and monitoring of food intake, and exclusion of acute nutritional deficiencies
(ii) Half-yearly internal medicine and nutritional controls until two years postsurgical, thereafter 12-month intervals
(iii) Gynaecological/obstetric provision is strongly recommended
(iv) In case of nutritional deficiencies, controls have to be intensified, especially when pregnancy is planned
(v) Surgical controls if necessary or any complications occur (as well as recommended three months after surgery)
Pregnancy control interval:
(i) Obstetric examination at regular intervals at least every 4–6 weeks with control of weight, urine, and blood pressure, and narrower control intervals if complications occur, decided on individual basis
(ii) Regular fetal growth control (check for SGA and LGA) every 4–6 weeks starting from 24th week of pregnancy. Further Doppler ultrasound examinations might be necessary
(iii) Internal medicine and nutritional controls every trimester
(iv) Explore nutrient uptake, and check full blood count, clinical chemistry, coagulation, vitamins A, D, E, K, B12, iron status, folic acid, parathyroid hormone and protein, albumin, A1c, glucose, and TSH at least every trimester
(v) Additional laboratory controls if possible: thiamine and zinc
(vi) If necessary, closer intervals have to be considered on an individual basis (2–4 weeks in case of deficiencies, which need to be corrected).
Immediate contact with an experienced surgeon in case of unexpected symptoms (especially gastrointestinal)
Immediate consultation in case of emergencies:
(i) Acute persistent abdominal pain → consult: gynaecologist/obstetrician and surgeon
(ii) Persistent vomiting (consider thiamine deficiency; see below sections) → consult gynaecologist/obstetrician, internal specialist, and surgeon
A close interdisciplinary cooperation is highly necessary to provide optimal pregnancy outcomes
Specialized centres with experience in the care of pregnant women after bariatric surgery need to be contacted or should fully take care of pregnancies after bariatric surgery
Drugs not allowed in pregnancy should be discontinued before pregnancy if possible or switched to drugs allowed in pregnancy (e.g., ACE inhibitors, statins, several glucose-lowering drugs). If this is not possible, risk assessment has to be performed in agreement with the patient

**Table 2:** Suggestions for diagnosis of gestational diabetes and overt diabetes in early pregnancy (<20 weeks of gestation) following metabolic surgery using capillary blood glucose monitoring (adapted from [40, 41]).

Fasting	≥95 mg/dl
1 h postprandially	In patients after gastric bypass/bariatric surgery of unknown significance (see text)
2 h postprandially	≥120 mg/dl
Overt diabetes diagnosis	
Fasting	≥126 mg/dl
HbA1c	≥6.5%
Random	In patients after gastric bypass/bariatric surgery of unknown significance (see text)

HbA1c values are applicable after bariatric surgery. Hypoglycaemia can also occur more than two hours after meal intake.

**Table 3:** Weight gain in pregnancy according to preconceptional BMI, adapted by IOM Guidelines 2009 [44].

BMI	BMI limit (kg/m^2^) (WHO)	Recommended weight gain in pregnancy (kg)	Recommended weight gain per week (2nd and 3rd trimesters)
Underweight	<18.5	13–18	0.5
Normal weight	18.5–24.9	11–16	0.5
Overweight	25.0–29.9	7–11	0.3
Obesity	≥30.0	5–9	0.2

**Table 4:** D-A-CH recommendations for supplementation of nutrients in pregnancies [68], tolerable upper intake levels according to EFSA [26] in pregnancy, and further nutritional recommendations in pregnancies after bariatric surgery according to Schultes et al. [25], Kaska et al. [75], Gonzalez et al. [28], Quyang et al. [7], Kushner et al. [62], ACOG [19], and Busetto et al. [5].

Nutrient	Recommended daily dietary intake during pregnancy (D-A-CH) [68]	UL (per day)	Pregnancy after bariatric surgery (per day)
Iron	30 mg	45 mg	100–200 mg [25], 40–65 mg [75], 65 mg [7], and 200 mg^8^ [5]
Calcium	1000 mg^6^	2500 mg	1500 mg [25], 1000–2000 mg [75], 1200–1500 mg [28], 1200 mg [7], and 1000–1200 mg [5]
Vitamin D	20 *µ*g = 800 IU^4^	100 *µ*g = 4000 IU^4^ [82]	400 IU [7], 1200–2000 IU [25], 2000–6000 IU [75], 1000 IU [5]
Vitamin A	1100 *µ*g equivalent^1^ from the 4th month	3000 *µ*g = 10000 IU	No more than 5000 IU^1^ [7, 75], 770 *µ*g [28]
Vitamin E	13 mg equivalent^2,3^	300 mg, 1000 mg	—
Vitamin K	60 *µ*g	—	120 *µ*g [7]
Vitamin B12	3.5 *µ*g	—	1000 *µ*g every 3 months i.m. [5, 25], 350 *µ*g orally/day or 1000 *µ*g every month [75], 1000 *µ*g/week i.m. or 350–500 *µ*g/day p.o. [7]
Folic acid	550 *µ*g	1 mg	600–800 *µ*g [19], 400 *µ*g [28], 800 *µ*g [7], 4 mg [75], 400 *µ*g or 5 mg^7^ [5]
Iodine	230 *µ*g	600 *µ*g, 1100 *µ*g	250 *µ*g [75], 200 *µ*g [28]
Zinc	10 mg	25 mg, 40 mg	11 mg [7], 20–30 mg [25], 15 mg [75]
Magnesium	310 mg	250 mg^5^, 350 mg^5^	200–1000 mg [75]

UL = upper limit; IU = international unit. In general, most of vitamins and trace elements mentioned are contained in typically available supplements used in pregnancy (e.g., Femibion, Pregnavit). ^1^1 mg retinol equivalent = 6 mg all-*trans*-*β*-carotene = 12 mg other provitamin A carotenoids = 1 mg retinol = 1.15 mg all-*trans*-retinyl acetate = 1.83 mg all-*trans*-retinyl palmitate; 1 IU = 0.3 *µ*g retinol. ^2^1 mg RRR-*α*-tocopherol equivalent = 1 mg RRR-*α*-tocopherol = 1.49 IU; 1 IE = 0.67 mg RRR-*α*-tocopherol = 1 mg all-rac-*α*-tocopheryl acetate. ^3^1 mg RRR-*α*-tocopherol (D-*α*-tocopherol) equivalent = 1.1 mg RRR-*α*-tocopheryl acetate (D-*α*-tocopheryl acetate) = 2 mg RRR-*β*-tocopherol (D-*β*-tocopherol) = 4 mg RRR-*γ*-tocopherol (D-*γ*-tocopherol) = 100 mg RRR-*δ*-tocopherol (D-*δ*-tocopherol) = 3.3 mg RRR-*α*-tocotrienol (D-*α*-tocotrienol) = 1.49 mg all-rac-*α*-tocopheryl acetate (D, L-*α*-tocopheryl acetate). ^4^1 *µ*g vitamin D = 40 IU. ^5^This UL does not include nutritional intake of magnesium from food or fluids and accounts for supplements only. ^6^1200 mg calcium in women <19 years of age. ^7^5 mg in patients with T2DM or BMI > 30 kg/m^2^ until 12 weeks of gestation. ^8^2–3 times daily. All NIH recommendations for women >18 years of age; i.m., intramuscular; p.o., per os.

**Table 5:** D-A-CH recommendations of nutritional intake during lactation [68] and tolerable upper intake level (UL) according to EFSA Guidelines [102] or the NIH [103] for healthy nonbariatric women as well as recommendations for intake after bariatric surgery for women (when available, data specific for lactation are reported).

Nutrient	Recommended daily dietary intake during lactation (D-A-CH reference)	UL per day	Recommended daily intake after bariatric surgery
Iron	20 mg	45 mg	45–60 mg [6, 62] up to 300 mg [104]
Calcium	1000 mg^6^	2500 mg	1200–1500 mg [6, 62], 1500–2000 mg RYGB [104], BPS/DS 1800–2400 mg [62, 104]
Vitamin D	20 *µ*g = 800 IU^4^	100 *µ*g = 4000 IU^4^ [82]	At least 3000 IU [6] up to 6000 IU [62]
Vitamin A	1500 *µ*g equivalent^1^	3000 *µ*g = 10000 IU	5000–10000 IU^9^ [62, 104]
Vitamin E	17 mg equivalent^2,3,7^	300 mg, 1000 mg [62]	Lactation 19 mg, else 15 mg [62]
Vitamin K	60 *µ*g	No recommendation	90–120 *µ*g, BPS 300 *µ*g [62]
Vitamin B12	4.0 *µ*g^8^	No recommendation	1000 mg/month i.m. or s.c. [6, 62]. 350–500 *µ*g p.o. [62, 104]
Folic acid	450 *µ*g	1 mg	400 *µ*g [6, 104], 400–800 *µ*g, 800–1000 *µ*g^10^ [62]
Iodine	260 *µ*g	600 *µ*g, 1100 *µ*g	—
Zinc	11 mg	25 mg, 40 mg	BPS 16–22 mg RYGB 8–22, mg, SG, LABG 8–11 mg [62]
Magnesium	390 mg	250 mg^5^, 350 mg^5^	—

^1^1 mg retinol equivalent = 6 mg all-*trans*-*β*-carotene = 12 mg other provitamin A carotenoids = 1 mg retinol = 1.15 mg all-*trans*-retinyl acetate = 1.83 mg all-*trans*-retinyl palmitate; 1 IU = 0.3 *µ*g retinol. ^2^1 mg RRR-*α*-tocopherol equivalent = 1 mg RRR-*α*-tocopherol = 1.49 IU; 1 IU = 0.67 mg RRR-*α*-tocopherol = 1 mg all-rac-*α*-tocopheryl acetate. ^3^1 mg RRR-*α*-tocopherol (D-*α*-tocopherol) equivalent = 1.1 mg RRR-*α*-tocopheryl acetate (D-*α*-tocopheryl acetate) = 2 mg RRR-*β*-tocopherol (D-*β*-tocopherol) = 4 mg RRR-*γ*-tocopherol (D-*γ*-tocopherol) = 100 mg RRR-*δ*-tocopherol (D-*δ*-tocopherol) = 3.3 mg RRR-*α*-tocotrienol (D-*α*-tocotrienol) = 1.49 mg all-rac-*α*-tocopheryl acetate (D, L-*α*-tocopheryl acetate). ^4^1 *µ*g vitamin D = 40 IU. ^5^The UL does not include magnesium from nutritional sources or fluid and accounts for supplements only. ^6^1200 mg calcium in women <19 years of age. ^7^Around 260 *µ*g RRR-*α*-tocopherol equivalent extra per 100 g secreted milk. ^8^Around 0.13 *µ*g vitamin B12 extra per 100 g secreted milk. ^9^Depending on procedure, LABG 5000 IU, RYGB or SG 5000–10000 IU, BPS 10000 IU per day, *β*-carotene form does not contribute to vitamin A toxicity. ^10^To women of childbearing age. All NIH recommendations for women >18 years of age; i.m., intramuscular; p.o., per os; BPS = biliopancreatic diversion; RYGB = Roux-en-Y gastric bypass; SG = sleeve gastrectomy; LAGB = laparoscopic gastric banding; DS = duodenal switch surgery.
